# Allele-specific transcription factor binding in a cellular model of orofacial clefting

**DOI:** 10.1038/s41598-022-05876-7

**Published:** 2022-02-02

**Authors:** Katharina L. M. Ruff, Ronja Hollstein, Julia Fazaal, Frederic Thieme, Jan Gehlen, Elisabeth Mangold, Michael Knapp, Julia Welzenbach, Kerstin U. Ludwig

**Affiliations:** 1 School of Medicine and University Hospital Bonn, Institute of Human Genetics, University of Bonn, Bonn, Germany; 2 Centre for Human Genetics, University of Marburg, Marburg, Germany; 3 Institute for Medical Biometry, Informatics and Epidemiology IMBIE, University of Bonn, Bonn, Germany

**Keywords:** Medical genetics, Genetics research

## Abstract

Non-syndromic cleft lip with/without cleft palate (nsCL/P) is a frequent congenital malformation with multifactorial etiology. While recent genome-wide association studies (GWAS) have identified several nsCL/P risk loci, the functional effects of the associated non-coding variants are largely unknown. Furthermore, additional risk loci remain undetected due to lack of power. As genetic variants might alter binding of transcription factors (TF), we here hypothesized that the integration of data from TF binding sites, expression analyses and nsCL/P GWAS might help to (i) identify functionally relevant variants at GWAS loci, and (ii) highlight novel risk variants that have been previously undetected. Analysing the craniofacial TF TFAP2A in human embryonic palatal mesenchyme (HEPM) cells, we identified 2845 TFAP2A ChIP-seq peaks, several of which were located near nsCL/P candidate genes (e.g. *MSX1* and *SPRY2*). Comparison with independent data suggest that 802 of them might be specific to craniofacial development, and genes near these peaks are enriched in processes relevant to nsCL/P. Integration with nsCL/P GWAS data, however, did not show robust evidence for co-localization of common nsCL/P risk variants with TFAP2A ChIP-seq peaks. This data set represents a new resource for the analyses of craniofacial processes, and similar approaches with additional cell lines and TFs could be applied to generate further insights into nsCL/P etiology.

## Introduction

Cleft lip with or without cleft palate (CL/P) is a frequent form of human orofacial clefting, and ranks among the most common of all congenital malformations^1^. In approximately one third of patients, CL/P occurs within the context of a known genetic syndrome^2,3^. However, in the majority of cases, CL/P arises as an isolated malformation, and is referred to as non-syndromic CL/P (nsCL/P)^3^. NsCL/P has a multifactorial etiology, which is characterized by the contribution of both genetic and environmental risk factors^4^. Heritability estimates from twin studies and multiplex pedigrees are high, with reported values of up to 90%^5,6^. This suggests that genetic factors make a substantial contribution to nsCL/P etiology.

Over the past decade, multiple investigations have been performed to identify the causal variants, risk genes, and functional mechanisms that contribute to nsCL/P^7^. These have included several genome-wide association studies (GWAS) and meta-analyses in diverse populations. Together, these genetic studies have identified 45 nsCL/P risk loci, which explain around 30% of the heritability^8–21^. Still, identifying causal variants at these loci remains challenging, since most of the associated single nucleotide polymorphisms (SNPs) are (i) located in non-coding regions, and (ii) their biological effect is difficult to dissect due to the presence of linkage disequilibrium^22^.

One of the mechanisms through which risk variants in non-coding regions can contribute to disease phenotypes is altered transcription factor (TF) binding to *cis*-regulatory elements^23^. Differential TF binding can modify the expression pattern of direct target genes, and also trigger downstream effects at the gene network level. Since the majority of gene regulation networks are highly cell-type and cell-state-specific, identifying effects of this nature requires analyses of cellular systems that are relevant to the specific disease in question^24^.

The major goal of the present study was to develop a framework for investigation of transcription factor binding events in nsCL/P, through integration of molecular data from human embryonic palatal mesenchyme (HEPM) cells^25–27^ and nsCL/P GWAS data. We first identified candidate TFs in HEPM through expression profiling. Among those, we prioritized TFAP2A for further analyses, for several reasons. First, deleterious mutations in *TFAP2A* cause Branchio-Oculo-Facial Syndrome^28^. This syndrome is characterized by dysmorphic anomalies and characteristic facial phenotypes including cleft palate^28^. Second, TFAP2A has been shown to bind to the nsCL/P risk variant rs642961, located within an enhancer of the nsCL/P candidate gene *IRF6*^20^. Furthermore, the TFAP2A-IRF6 pathway is a well-established pathway involved in orofacial clefting^29^. Finally, *TFAP2A* is located at an nsCL/P risk locus (6p24) previously identified by GWAS^8^.

Performing chromatin immunoprecipitation followed by sequencing (ChIP-seq) in HEPM, we then identified ChIP-seq peaks indicative of TFAP2A binding and confirmed plausibility of these regions through comparisons with external data sets. This map of binding regions was next integrated with (i) genotype information from HEPM cells, and (ii) summary statistics of a previous nsCL/P GWAS meta-analysis^9^ with two aims. First, we wanted to identify potential causal variants at known GWAS risk loci. Therefore, we looked whether TFAP2A ChIP-seq peaks co-localize with associated risk variants, and whether they exhibit allele-specific TFAP2A binding effects at heterozygous positions. Second, we hypothesized that binding of TFAP2A in HEPM might contribute to nsCL/P at loci that are not yet genome-wide significant, due to limited power of current GWAS studies. While the results of our analyses provide only limited evidence for a role of genetically mediated effect of nsCL/P risk variants at TFAP2A ChIP-seq peaks in HEPM, the map of binding sites as well as the framework described here can be used as blueprint for further integrative analyses of epigenetic and genetic data in nsCL/P.

## Results

### *TFAP2A* is a candidate TF expressed in HEPM cells

**Figure 1:**
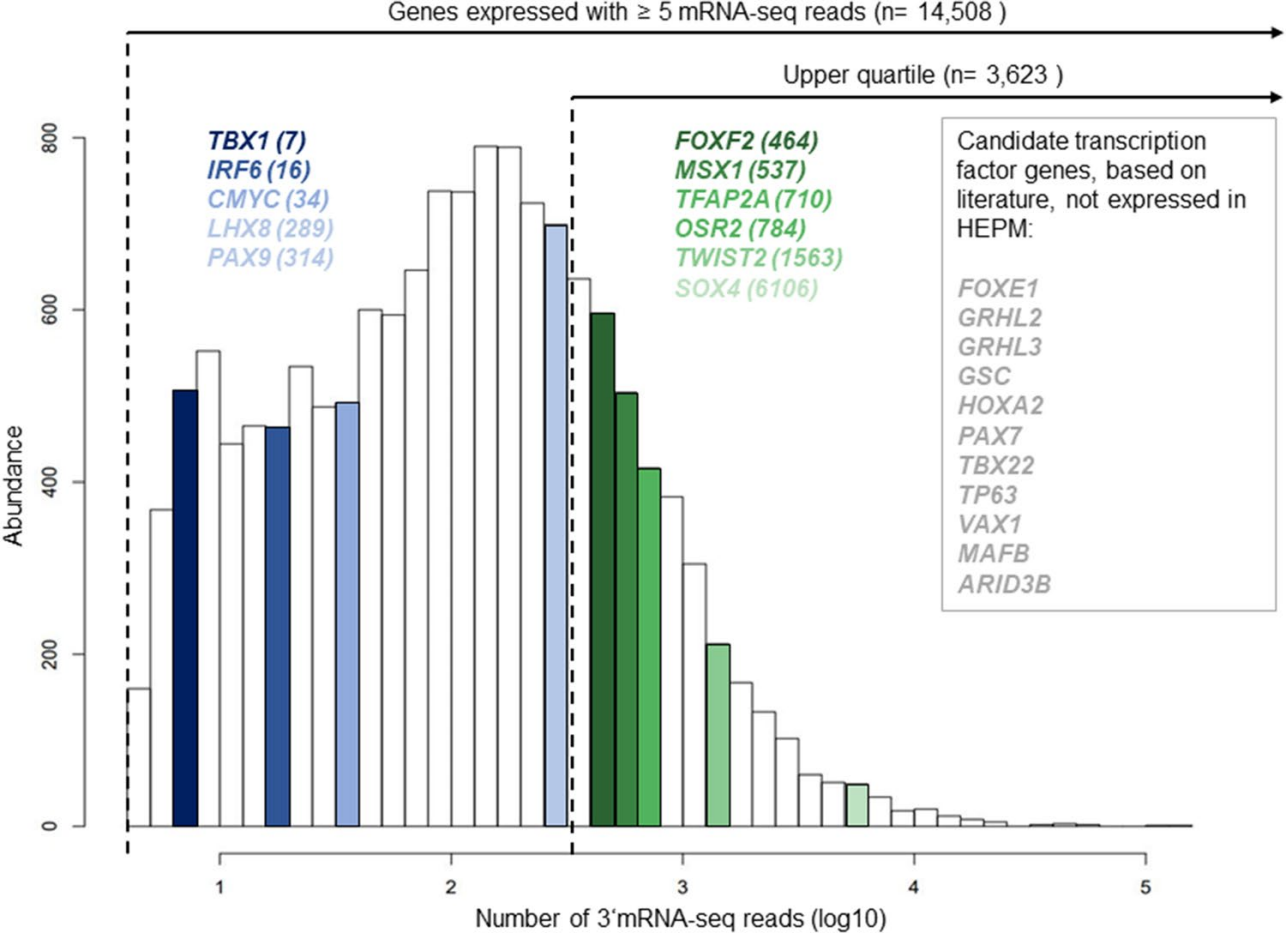
Expression analysis in human embryonal palatal mesenchyme (HEPM) cells. Density plot of 3′mRNA sequence reads, provided at log10 scale for the average of three replicates. In total, 14,508 genes were observed with ≥ 5 reads, and 3,623 genes were in the upper quartile (≥ 327 reads) of all genes with ≥ 5 reads. Out of 22 candidate transcription factors for craniofacial development, 11 were expressed in HEPM.

RNA-seq in HEPM revealed 14,508 expressed genes, defined by an expression with ≥ 5 aligned m-RNA-seq reads in average. This included 350 TFs that were represented by motifs in the JASPAR core vertebrate assembly 2020^30^ (Supplementary Table S1, Fig. 1). In addition, literature research revealed 22 TFs that have been previously reported with a role in craniofacial development and/or orofacial clefting (Supplementary Table S2). Integration both data sets revealed an overlap of 11 TFs, with 6 TFs being considered “strongly expressed” as defined by the largest quartile (Supplementary Table S2, Fig. 1). For 2 out of these 11 TFs (*TFAP2A* and *MSX1*), additional support for an involvement in nsCL/P etiology was available through their location at previously identified nsCL/P risk loci (i.e., 6p24 and 4p16^8^). In the present study we prioritized TFAP2A, as the GWAS signal at this locus was mainly driven by the European population, thus matching the ethnicity of the HEPM donor (as confirmed by principal component analysis of array-based genotypes, *data not shown*).

### TFAP2A ChIP-seq peaks are located near nsCL/P candidate genes

**Figure 2:**
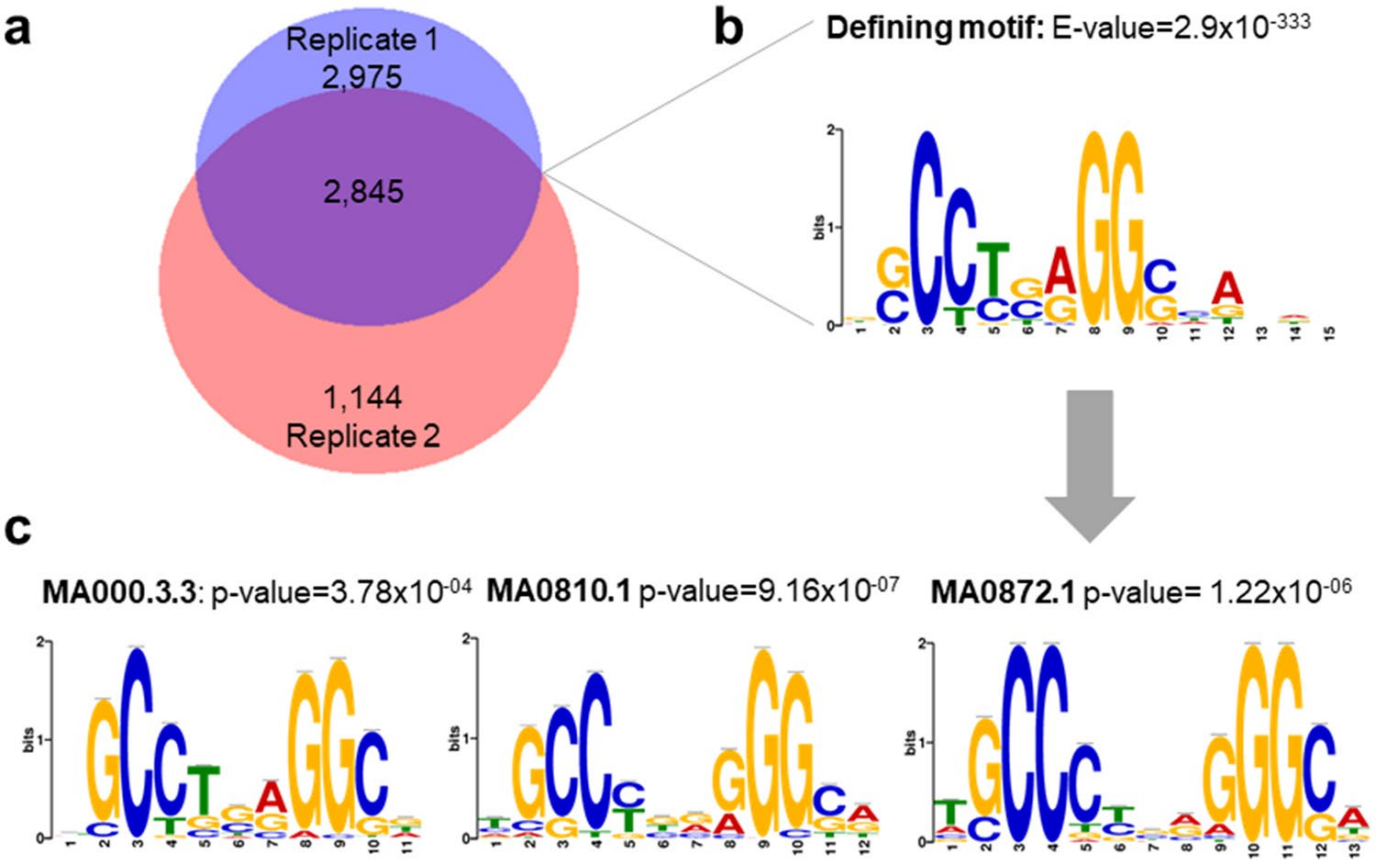
TFAP2A high confidence peaks (hc-peaks) in human embryonal palatal mesenchyme (HEPM) cells (**a**) Venn diagram showing the total number of TFAP2A ChIP-seq peaks across two replicates (replicate 1 n = 5820; replicate 2 n = 3989). The overlap of 2845 regions was denoted as „hc-peaks” for the subsequent analyses. Plotted with BioVenn^©^ (2020). (**b**) Identification of the most abundant binding motif within hc-peaks (present in n = 1535 hc-peaks) with MEME-ChIP. (**c**) Comparative analysis using Tomtom and JASPAR core 2018 assembly identified the significant alignment of the enriched motif with three distinct TFAP2A binding motifs.

**Figure 3:**
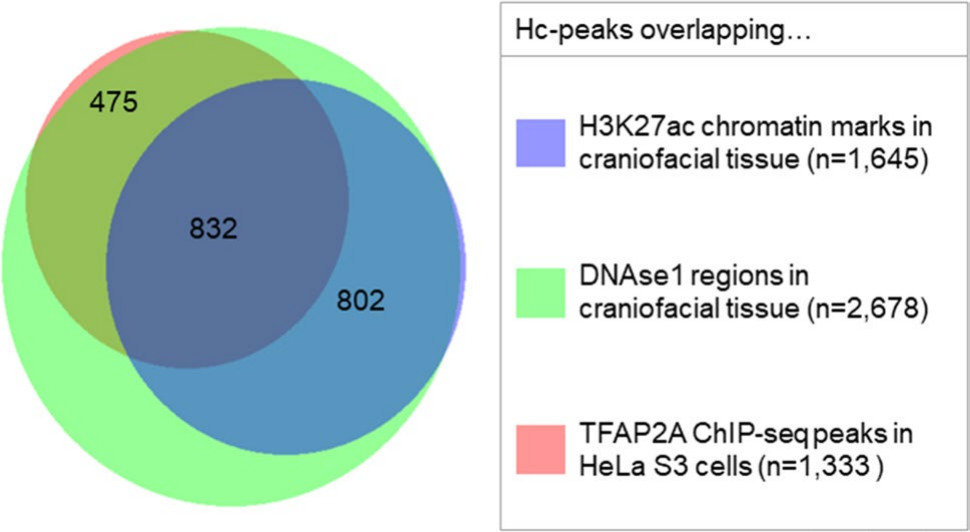
Intersection of TFAP2A hc-peaks overlapping with TFAP2A ChIP-seq peaks in HeLa S3 and/or chromatin marks in craniofacial tissue. Venn diagram displaying the intersection of 2,713 hc-peaks overlapping with TFAP2A ChIP-seq peaks in HeLa S3/ DNase1 hypersensitivity regions/ H3K27ac chromatin marks in CS15 craniofacial tissue. Hc-peaks that did not overlap with any of these are not displayed in the diagram (n = 132). A total of 802 hc-peaks overlapped with DNAse1 hypersensitivity regions and H3K27ac marks in CS15 craniofacial tissue, but did not overlap with a TFAP2A ChIP-seq peak in HeLa S3 cells, thus indicating craniofacial-specific hc-peaks. Overlaps were defined as described in the Methods. Plotted with BioVenn^©^ (2020).

Peak calling of the TFAP2A ChIP-seq reads in two replicates revealed 5,820 and 3,989 unique peaks, respectively (FDR < 5%, fold enrichment (FE) 5–50, Supplementary Tables S3, S4). The intersection encompassed 2845 regions, which were considered high confidence peaks (hc-peaks, average size of 324 bp, Supplementary Table S5, Fig. 2a). Retrieving the sequence from the hc-peak summit regions identified a highly enriched 15 bp motif (e-value = 2.9 × 10^−333^; present in 1535 summit regions Fig. 2b), which matched the three TFAP2A binding motifs of the JASPAR core 2018 assembly^31^ (Fig. 2c). We also compared the TFAP2A ChIP-seq peaks from HEPM cells to a set of TFAP2A ChIP-seq peaks obtained from HeLa S3 cells^32^, which is a non-craniofacial cell line of human cervical cancer cells^33^. The purpose of using data from HeLa S3 cells in our study was to use them as background to identify TFAP2A ChIP-seq peaks that might be specific to facial mesenchyme based on their absence in HeLa S3 cells. We observed a highly significant co-localization, with 1333 of the 2845 hc-peaks overlapping at a minimum of one site (*P*_χ2_ < 0.0001, Supplementary Table S5, Fig. 3), indicative of plausibility of the ChIP-seq peaks in HEPM. The assignment of genes adjacent to hc-peaks using GREAT yielded a total of 3470 genes which can be considered candidates for TFAP2A-mediated regulation (Supplementary Table S5). These genes included a set of 10 established nsCL/P candidate genes, such as *MSX1*^8^, *TP63*^12^, and *SPRY2*^11^ (Table 1), eight of which were also expressed in HEPM cells (average of ≥ 5 reads, Table 1).

**Table 1:** Candidate genes for nsCL/P located near TFAP2A binding sites in HEPM cells.

Hc-peak information			NsCL/P candidate gene^a^			
Chromosome	Start	End	Gene symbol	Distance peak to TSS (bp)	Reference (PubMed-ID)	Expression in HEPM cells (average no. of 3′RNA-seq reads)^b^
1	94,787,207	94,787,445	*ARHGAP29*	− 84,205	Beaty et al. 2010 (PMID: 20,436,469)	1436
1	94,791,145	94,791,437		− 88,170		
3	99,844,546	99,844,795	*FILIP1L*	− 11,314	Beaty et al.2013 (PMID: 23,512,105)	523
3	99,878,493	99,878,721		45,250		
3	189,281,805	189,282,039	*TP63*	− 67,294	Leslie et al. 2017 (PMID: 28,054,174)	Not expressed
3	189,655,227	189,655,963		306,379		
4	4,860,996	4,861,363	*MSX1*	− 213	Yu et al. 2017 (PMID: 28,232,668)	537
13	80,205,476	80,205,754	*SPRY2*	708,179	Ludwig et al. 2012 (PMID: 22,863,734)	125
13	80,604,734	80,605,110		308,872		
13	80,788,600	80,788,933		125,027		
13	80,915,560	80,915,801		− 1887		
15	32,962,254	32,962,778	*GREM1*	− 47,659	Ludwig et al. 2016 (PMID: 26,968,009)	5471
15	62,898,345	62,898,659	*TPM1*	− 436,382	Ludwig et al. 2012 (PMID: 22,863,734)	7613
15	63,233,187	63,233,480		− 101,550		
15	74,838,564	74,838,823	*ARID3B*	5176	Ludwig et al. 2017 (PMID: 28,087,736)	4
16	4,166,662	4,166,962	*ADCY9*	− 626	Sun et al. 2015 (PMID: 25,775,280)	126
17	54,240,701	54,240,761	*NOG*	− 430,329	Mangold et al. 2010 (PMID: 20,023,658); Leslie et al. 2015 (PMID:25,704,602)	60
17	54,672,130	54,672,266		1138		

^a^NsCL/P candidate genes as putative downstream target genes of hc-peaks assigned with GREAT (association rules described in methods).

^b^Average number of 3′-mRNA-seq reads (Supplementary Table S1). Methods provided in the text.

### Developmental processes are enriched at sites of TFAP2A ChIP-seq peaks in HEPM

**Figure 4:**
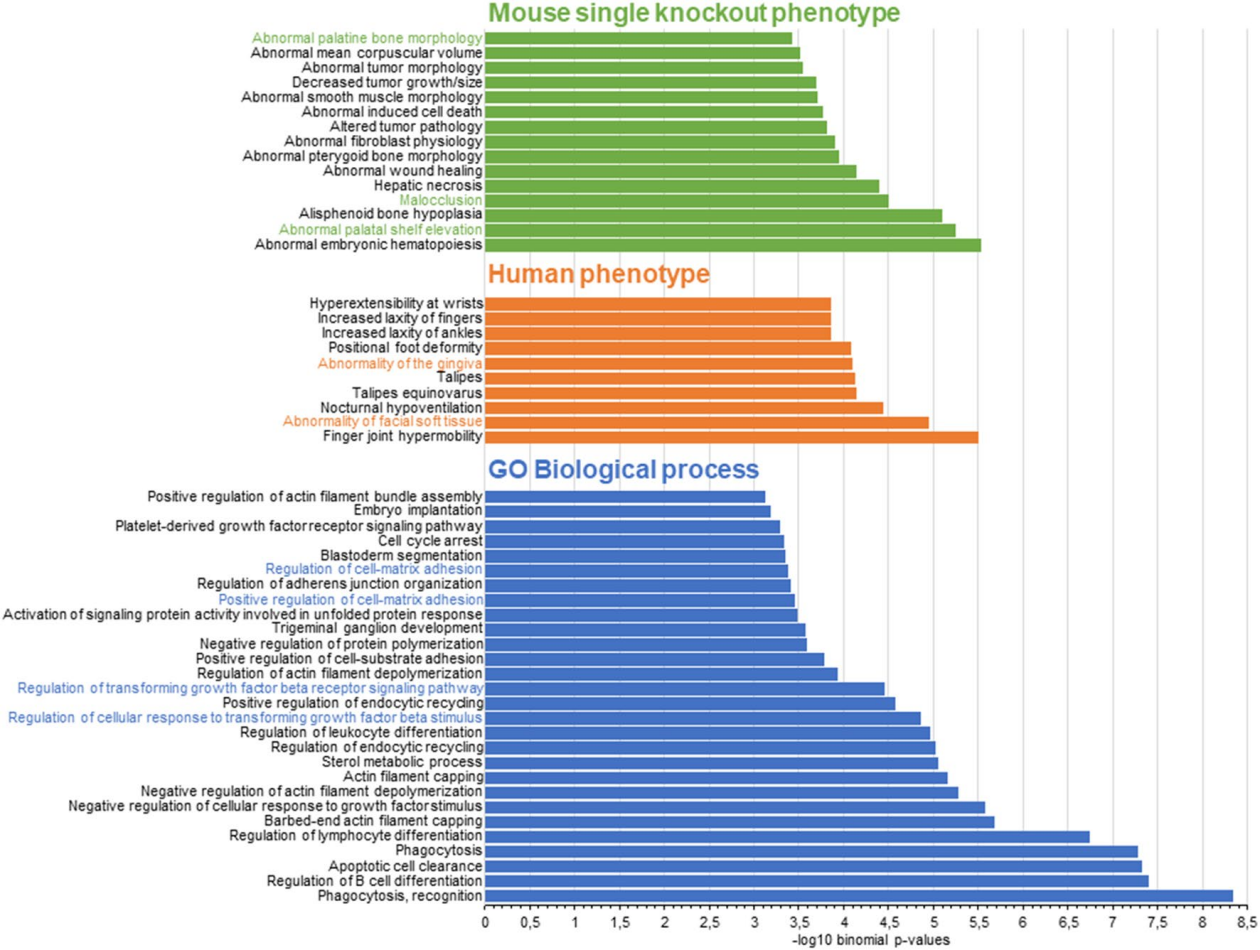
Gene Ontology (GO) analysis of 802 craniofacial-specific TFAP2A high confidence peaks (hc-peaks). Bar charts displaying -log10 binomial p-values of enriched mouse single knockout phenotypes with false discovery rate (FDR) < 0.05, human phenotypes with FDR < 0.05 and GO biological processes with FDR < 0.05. Terms relating to orofacial clefting are highlighted in color. Genes associated with these terms in assigned to TFAP2A hc-peaks, based on their proximity, as described in the Methods, are displayed in the boxes.

We next investigated the relevance of the HEPM-based TFAP2A ChIP-seq peaks for craniofacial development. Using previously published DNase1 hypersensitivity regions from embryonic craniofacial tissue of Carnegie Stage 15^34^, we identified co-localization of 2678 out of 2845 hc-peaks (*P*_χ2_ < 0.0001, Supplementary Table S5, Fig. 3). Of these, 1,634 (61%) also overlapped with at least 1 signal for H3K27ac in CS15 craniofacial tissue, which is suggestive of enhancer activity of this region during human craniofacial development (*P*_χ2_ < 0.0001, Supplementary Table S5, Fig. 3). Interestingly, 802 of the 2845 hc-peaks overlapped with both DNase1 and H3K27ac marks in CS15 craniofacial tissue, but did not overlap with the TFAP2A ChIP-seq peaks in HeLa S3 cells (Supplementary Table S5, Fig. 3). Results of a GO analysis for genes located at these 802 hc-peaks yielded significant results for 28 biological processes, 10 human phenotypes, and 15 mouse single knockout phenotypes with an FDR *q*-value < 0.05 (Supplementary Tables S6–S8, Fig. 4). These included processes such as “regulation of transforming growth factor beta receptor signaling pathway” or “regulation of cell–matrix adhesion”, but also “abnormality of facial soft tissue” and “abnormal palatine bone morphology”. When comparing these GO enrichment results with those obtained for all 2845 hc-peaks, we found that several terms were either more significantly enriched, or did only show an association in the analysis of craniofacial-specific peaks (Supplementary Tables S6–S11). Together, these findings suggest that TFAP2A-binding in human palatal mesenchymal cells might play a role in craniofacial processes.

### Limited evidence for GWAS risk variants in TFAP2A peaks

Subsequent positional integration of the 2845 hc-peaks with the topological associating domains (TAD) comprising the 45 nsCL/P GWAS risk loci^21,35^ revealed the presence of 70 TFAP2A hc-peaks within 20 of these TADs. Among the 46 common SNPs located within these hc-peaks, we observed rs672819 at 1q32.1 to be in moderate LD with rs3753517 (D′ = 0.51; r^2^ = 0.18), which is the lead variant at this locus, and of rs447476 with rs2303914, the lead SNP at 2p25.1 (D′ = 0.91; r^2^ = 0.28)^21^ (Supplementary Table S12). At genome-wide scale, we observed a total of 1,564 common SNPs being located within hc-peaks (Supplementary Table S13). Data integration with a previously published nsCL/P GWAS meta-analysis ^9^ revealed 29 SNPs which had *P*_GWAS_ < 0.01 (including four SNPs at *P*_GWAS_ < 0.001: rs463271 (22q11.21, *P*_GWAS_ = 2.72 × 10^−4^); rs12882215 and rs7144455 (14q24.3, *P*_GWAS_ = 3.30 × 10^−4^ and *P*_GWAS_ = 3.77 × 10^−4^, respectively, D′ = 1.0; r^2^ = 0.85); and rs4774822 (15q21.3; *P*_GWAS_ = 6.27 × 10^−4^, Supplementary Table S13). Together, our data provide only very limited support for the hypothesis that the association of common risk SNPs at nsCL/P GWAS loci can be attributed to TFAP2A binding in HEPM.

### Identification of candidate variants for nsCL/P with allele-specific effects on binding

We finally investigated whether there is evidence for a genetically-mediated effect of TFAP2A-bound regions (and variants therein) outside of the known GWAS loci, for instance, at suggestive loci. For the analysis of allele-specific effects of TFAP2A binding, we first retrieved array-based genotypes for HEPM and found that 461/1,564 SNPs were heterozygous, including rs463271, rs672819 and rs447476 (see above). The other 3 SNPs at *P*_GWAS_ < 0.001 were found to be homozygous. At 18 positions we found statistical evidence for allele-specific reads (binomial *p* < 0.05, with consistent directions of effect across both replicates, Supplementary Table S14), but this did neither include rs463271 nor any other SNP with statistically significant association results from the nsCL/P GWAS meta-analysis (lowest *P*-value observed: *P*_GWAS_ = 0.091 for rs616822 at 18q21.33). Analysis of potential downstream genes at these 18 sites revealed that 8 and 10 SNPs were associated with 1 or 2 possible target genes, respectively (Supplementary Table S14). However, none of these genes has been reported as candidate gene for nsCL/P, and also GO enrichment for these gene sets did not yield any significant process at an FDR *q*-value < 0.05.

## Discussion

A major challenge to our understanding of the biological role of nsCL/P-associated risk variants is their predominant mapping to non-coding regions of the genome. Although in vivo and in vitro approaches exist to identify functional variants among the statistically associated passenger variants, the major prerequisite is the analysis of disease-relevant tissue. At mechanistic level, altered TF binding to variant alleles has been suggested as one biological process through which non-coding risk variants might contribute to complex traits and diseases, including nsCL/P^22,25^. Examples include a recent study by Huo et al.^36^, who analyzed TFBS from ChIP-seq data of 30 TFs in brain tissues. The authors identified 132 schizophrenia risk variants that exert a functional effect on gene expression by disrupting the binding sites of 21 TFs. Also, Benaglio et al.^37^ investigated the regulatory landscape and gene expression profiles of human induced pluripotent stem cell derived cardiomyocytes and identified differential binding of the cardiac TF NKX2-5 to risk SNPs at GWAS loci for electocardiographic traits such as atrial fibrillation.

In the present study we examined binding of TFAP2A in human embryonal palatal mesenchyme cells which serve as in vitro model of human palate development, since they have been retrieved from the palatal shelves of an embryo at a gestational age when elevation and fusion processes take place^27^. TFAP2A was chosen since it is (i) encoded by an nsCL/P candidate gene with robust evidence for an involvement in craniofacial development, and (ii) is strongly expressed in HEPM. Further evidence for a role of TFAP2A has been gained in mice, where knock-down of *Tfap2a* leads to severe malformations including facial clefting^24,38^, and in a multi-omics study of human dental pulp stem cells^39^. Importantly, while the orchestrational role of TFAP2A in structures derived from neural crest or ectodermal cells is well documented, few data are available concerning its role in the palatal mesenchyme^40,41^. For instance, it has been shown that in mice, *Tfap2a* is expressed in the facial mesenchyme during palate development^42^, and knock down of *Tfap2a* leads to an increase in the expression of *Fgf8,* which is implicated in the differentiation of mesenchymal cells into cartilage in the anterior plate^43,44^. While this suggests a regulatory connection of *Tfap2a* and *Fgf8*, in our study, we did not observe any TFAP2A ChIP-seq peak in the proximity of *FGF8*. However, we observed TFAP2A binding sites near other nsCL/P candidate genes, such as *MSX1* on chr4p16^8^, and *SPRY2* on chr13q31^11^. At the *MSX1* locus, the TFAP2A ChIP-seq peak is located ~ 200 bp upstream of the *MSX1* transcription start site (TSS). In concordance with prior evidence of *Msx1* being expressed in the palatal mesenchyme at various stages of development, we also observed strong expression of *MSX1* in HEPM. Additionally, *Msx1* has been suggested to be required for normal outgrowth of palatal shelves and mesenchymal proliferation^45,46^, and research has shown that Tfap2a regulates *Msx1* expression in murine neural crest cells^47^. At the *SPRY2*-locus, several TFAP2A ChIP-seq peaks have been identified, the closest of which is located ~ 1.9 kb upstream of the TSS of *SPRY2*^11^. *Spry2* is expressed in mouse palatal mesenchyme, and both knock out and overexpression of the gene lead to cleft palate^48,49^. *Spry2* knock out mice display an increased proliferation of palatal mesenchyme and a higher expression of other clefting genes, such as *Msx1*, *Etf,5* and *Ptx1*^48^. We also observed strong expression of *SPRY2* in HEPM. Together, these lines of evidence suggest that these three nsCL/P risk genes (*MSX1, SPRY2,* and *TFAP2A*) might form an nsCL/P regulatory network in HEPM cells. In addition, TFAP2A binding sites have been observed near other nsCL/P candidate genes from GWAS, i.e., *ARHGAP29, FILIP1L, TP63, GREM1, TPM1, ARID3B, ADCY9,* and *NOG.* With the exception of *TP63,* all of these were also expressed in HEPM. We also observed TFAP2A ChIP-seq peaks downstream of *CD58* (~ 16 kb), and *PTGS2* (two peaks; located ~ 261 bp and ~ 75 kb upstream), both of which have been implicated in the TFAP2A-gene regulatory network described by Razaghi-Moghadam et al.^39^. Together, these genes represent interesting candidates for further studies of nsCL/P in HEPM cells. Based on the well-established interaction between *TFAP2A* and *IRF6*, we specifically looked at TFAP2A binding sites near *IRF6*. We could not detect any TFAP2A ChIP-seq peaks within 1 Mb of the TSS of *IRF6*, and also *IRF6* expression was only merely detected above background in HEPM. Thus, while there is robust interaction of *TFAP2A* and *IRF6* in epithelial cells^29^, our data indicate that such effects might not be present in palatal mesenchymal cells. Importantly, in the present study, the genes were assigned to the hc-peaks on the basis of proximity. Therefore, long-distance regulatory effects of TFAP2A binding sites would have been missed by design.

In the comparison of TFAP2A ChIP-seq peaks in HEPM cells and HeLa S3 cells, almost half of the ChIP-seq peaks in HEPM cells were found to overlap between both cell lines. Besides indicating that these peaks are true positives, this finding also suggests that some TFBS are specific to HEPM cells (and, presumably, to craniofacial processes). In support of this we observed a strong overlap of TFAP2A sites within DNase1 hypersensitivity sites (94.1% overlap), and H3K27ac signals (57.8% overlap) from human embryonic craniofacial tissue. This indicates that these TFAP2A ChIP-seq peaks reside at chromatin sites that are accessible to TF binding during human facial development. The output of our GO analysis provides further support for a role of TFAP2A binding sites in facial development, as craniofacial-specific terms such as “abnormality of facial soft tissue”, “abnormal palatine bone morphology”, or “abnormal palatal shelf elevation” were significantly enriched. We also found evidence for a contribution of the transforming growth factor beta pathway in HEPM, which is implicated in the epithelial-mesenchymal-transition processes that occur during secondary palate formation^50,51^.

Finally, our data set was used to analyze whether common nsCL/P risk alleles located at the TFAP2A ChIP-seq peaks in HEPM might contribute to disease pathomechanism. We did not observe any risk SNP at test-wide significance within the TFAP2A ChIP-seq peaks. Four SNPs were detected at suggestive significance, which does not exceed the number expected by chance. In addition, no allele-specific effect was observed for the one variant of those four that was heterozygous. Further analyses outside of established GWAS risk loci identified 18 SNPs with allele-specific TFAP2A binding, but again, none of them showed a nominally significant association with nsCL/P. Thus, our data do not provide evidence that the biological effect at any of the common risk variants is genetically mediated through differential TFAP2A binding in mesenchymal cells.

Our study is influenced by some limitations. First, we investigated a two-dimensional in vitro model, which lacks the complexity of cell-to-cell interactions. Particularly, we might have missed effects that are driven by the interaction between epithelial and mesenchymal cells, and/or environmental clues, such as those present in three-dimensional embryonic palate^50–52^. This could be overcome by future investigations of three-dimensional organoid systems, such as recently established by Wolf et al.^53^ and Hughes et al.^54^. Second, the integration of TFAP2A binding peaks and genetic risk variants from GWAS meta-analyses only informs about common risk variants, but does not provide information on potential effects of rare variants on TFAP2A binding. Herefore, the integration of whole genome sequencing data would be required, e.g. those recently described by Bishop et al.^55^. Also, the analyses of allele-specific effects were limited by the fact that only one HEPM-donor was available, resulting in a limited number of heterozygous sites usable for the analysis. Finally, our approach does not cover effects of (i) TFAP2A in other cell types (e.g. oral facial epithelium), (ii) other TFs in HEPM (e.g. MSX1, which we also observed at high expression in HEPM), or (iii) other types of gene regulation (e.g. miRNA^56,57^). While potential TFs can be identified as presented in this study, alternative approaches also exist—for instance, integrating GWAS risk SNPs and TF databases such as JASPAR^30^. This, however, would then require subsequent identification of the relevant cell type for in vitro investigation, which is still a challenge for embryonic human phenotypes.

Taken together, the present data suggest that TFAP2A binding in HEPM might play a role in normal craniofacial development, and indicate a set of presumably craniofacial-specific TFAP2A ChIP-seq peaks near nsCL/P candidate genes, which might be functionally followed up. No strong evidence was obtained for the hypothesis that genetic variability at these sites contributes to nsCL/P etiology. Despite this, similar analyses in other cell types of relevance to craniofacial development might provide novel insights into our understanding of genetically-mediated nsCL/P risk.

## Methods

### Cell culture

A human embryonic palatal mesenchyme cell line was commercially available at ATCC (ATCC Cat# CRL-1486). Upon purchase these cells were cultured in DMEM-Dulbecco's Modified Eagle Medium (high Glucose) with 10% heat inactivated fetal bovine serum (FBS) and Penicillin/Streptomycin at a final concentration of 1%. Cells were stored in an incubator (37 °C, 5% CO_2_), with a change of medium every two days. After five days of cultivation, the cells were split using 0.25% Trypsin/EDTA. DNA and RNA were extracted from the cells using the DNeasy® Blood &Tissue kit and the RNaeasy® kit (QIAGEN, Germany), respectively, in accordance with the manufacturer’s protocols.

### RNA-Seq

To capture the expression profile of HEPM cells, 3’mRNA-Seq was performed in triplicate. For library preparation, the QuantSeq 3' mRNA-Seq Library Prep Kit (Lexogen, Austria) was used, in accordance with the manufacturer’s instructions. Sequencing was performed at 1 × 50 bp on an Illumina HiSeq2500, with a minimum depth of ~ 20 mio reads per sample. Reads were quality checked using FastQC (v0.11.7), adapters were trimmed using bbduk (BBMap v37.44), and reads were aligned to the GRCh37/hg19 reference genome using STAR Aligner (v2.5.2b). Gene expression was quantified using featureCounts (v1.5.1), and the Ensembl Human GRCh37.p13 annotation as a reference. Quality control was carried out using MultiQC (v1.2). Genes were classified as “expressed” if the average number of aligned mRNA reads was ≥ 5, and “strongly expressed” if the average number of aligned mRNA reads was within the upper quartile of all genes expressed with ≥ 5 mRNA-seq reads in HEPM (i.e., ≥ 327 reads). The JASPAR CORE vertebrate assembly (2020)^30^ was used to identify TFs among genes that are expressed in HEPM.

### Selection of candidate transcription factors

To identify TFs with an involvement in craniofacial development and/or orofacial clefting, a systematic search was performed in the Pubmed database. A TF was considered to be a candidate TF if the respective gene had been reported previously: (i) as a candidate gene at a nsCL/P risk locus; (ii) in a mutated state in patients with craniofacial malformations; (iii) to result in disturbed craniofacial development when modified in animal models; or (iv) to be part of a gene regulatory network involved in facial development. To identify TFs whose potential role in nsCL/P etiology involved a change of TF binding in HEPM, this list of candidate TFs was cross-referenced with the HEPM expression data.

### Genotyping

To determine genotypes for common variants in HEPM, DNA was extracted from HEPM cells. The DNA was then genotyped on an Illumina Infinium GSAv2.0 array (Illumina, USA), comprising ~ 700.000 SNPs with a major allele frequency > 0.1%. After stringent quality control, genotypes of SNPs that were not represented on the array were imputed using IMPUTE2 (v2.3.2), the 1000 genomes phase 3 GRCh37/hg19 variants as a reference panel, and an info metric threshold of 0.5. For imputed variants with mono-allelic binding in the subsequent ChIP-seq analysis, genotypes were validated by Sanger sequencing in order to exclude imputation artifacts.

### Chromatin Immunoprecipitation (ChIP-seq)

The SimpleChIP®Enzymatic Chromatin IP Kit (#9003, Cell Signaling Technology®, USA) was applied with minor modifications and using two replicates. Briefly, for each immunoprecipitation (IP), around 4 × 10^6^ HEPM cells were crosslinked for 10 min at room temperature using 37% formaldehyde at a final concentration of 1%. The reaction was stopped with 0.125 mM Glycine. Cells were washed twice with ice-cold PBS and scraped into a tube. Cells lysis was performed by sequential cold incubation (4 °C, on ice) with two buffers provided in the reagent kit. Chromatin was digested by 0.5 µl micrococcal nuclease per IP for 15 min at 37 °C on a constantly shaking heating block. After stopping digestion by the addition of 0.5 M EDTA, nuclei were suspended in ChIP buffer and sonicated with a Diagenode Bioruptor (settings: 50 cycles, 30 s sonication/30 s break). A fraction of the sample was used for measuring DNA-concentration using Nano Drop and to check fragmentation size via electrophoresis. A total of 0.5 µg DNA was used as the input control. A total of 0.005 µg of polyclonal ChIP Grade TFAP2A antibody (Abcam Cat# ab52222) was added to 25 µg of DNA and incubated overnight with rotation at 4 °C. Fragments were pulled down using Protein G magnetic beads, and then removed from the beads via incubation with ChIP elution buffer on a shaking thermomixer at 65 °C for 30 min. The supernatant was treated overnight with NaCl and Proteinase K in order to reverse DNA-crosslinking, and then purified using spin column tubes, as provided in the reagent kit.

### Library preparation and next generation sequencing

Library preparation for sequencing was performed using the NEBNext® Ultra™ II DNA Library Prep Kit for Illumina® (New England Biolabs® GmbH, USA), in accordance with the manufacturer’s protocol. AmPure XP beads (Beckman Coulter™, USA) were used for the cleanup steps. Since the amount of input DNA was < 50 ng, no size selection was performed. Equimolar pooling of the samples was then performed, and the quality of the library was controlled on an Agilent High Sensitivity D1000 system. The samples were diluted to a final concentration of 2 nM. To achieve sufficient coverage, each replicate was sequenced twice on an Illumina MiSeq v2. This yielded ~ 20 million 2 × 250 bp paired end reads for each sample (ChIP and input control), in accordance with the ChIP-seq guidelines of the ENCODE consortium^58^.

### Bioinformatic processing of ChIP-Seq data

Quality control of the fastq-files was performed using FastQC (v0.11.7). Adapter sequences were cut-off using Cutadapt (v1.15), and reads were trimmed to a maximum length of 200 bp. For each sample and replicate, fastq files of both sequencing runs were merged and aligned to the GRCh37/hg19 reference genome using Bowtie2 (v2.3.4). Peak calling was performed using MACS2 (v2.1.1). Quality control included the retention of peaks with a fold enrichment (FE) of 5–50, and a false discovery rate (FDR) < 0.05. Peaks mapping to irregular chromosomes and ENCODE blacklist regions were removed^59^ using BEDtools (v2.27.0). Peaks were then visually inspected in the Integrative Genomics Viewer (IGV, v.2.4.6). To extract a high-confidence set of peaks (termed “hc-peaks”), only those regions included in both replicates were included in the analysis.

### Motif discovery

Motif discovery was performed using the genomic sequence around the summit of each hc-peak (± 50 bp) and MEME ChIP (v.5.1.0). The similarity between TFAP2A motifs from the JASPAR Core 2018 assembly^31^ and the most significant motif from ChIP-seq was further quantified and displayed by presenting the optimal alignments with the Tomtom motif comparison tool (v.5.1.0).

### Comparison with other data sets

First, hc-peak positions were compared to TFBS in another TFAP2A ChIP-seq data set that had been obtained in HeLa S3 cells by ENCODE^32^. Here, concordant regions were defined as those for which HEPM TFAP2A ChIP-seq peaks showed a ≥ 50% overlap with the base pairs of HeLa S3 TFAP2A sites. Second, HEPM TFAP2A ChIP-seq peaks were compared with H3K27ac histone marks and DNase1 hypersensitivity sites derived from craniofacial tissues (Epigenomic Atlas of Human Craniofacial Development) at Carnegie stage 15 (CS15)^34^, indicating active regulatory elements in a human developmental stage that equated with the time point of the HEPM cells during craniofacial development. Since the ChIP-seq peak distribution for TFs and histone modifications differs, overlaps were defined using separate cut-offs (TF: overlap defined as 50% overlap; histone modifications: overlap defined as 1 bp). For both data sets, enrichment of the identified hc-peaks was determined using Chi2-test (1df).

### Allele-specific binding

NsCL/P associated SNPs that were located within the hc-peaks and predicted to be in a heterozygous state in the HEPM cells were extracted, and corresponding allele counts from ChIP-seq were analyzed using ABC (v.1.3). SNPs were considered allele-specific variants if one of the two alleles was overrepresented at a statistically significant level (*P* binomial < 0.05). The subset of hc-peaks that contained SNPs with allele-specific TF binding was compared to TFAP2A ChIP-seq peaks in HeLa S3 cells^32^ and chromatin marks in CS15 craniofacial tissue^34^.

### Gene ontology analysis

Gene Ontology (GO) enrichment analysis was performed using the Genomic Regions Enrichment of Annotations Tool (GREAT, v4.0.4) with default parameters (5 kb upstream, 1 kb downstream, and 1 Mb maximum extension with inclusion of curated regulatory domains). GO biological processes, human phenotypes, and mouse single knockout phenotypes were considered. For these analyses, three subsets were defined: (i) all hc-peaks; (ii) hc-peaks that contained SNPs with allele-specific effects; and (iii) hc-peaks that overlapped with H3K27ac markers and DNase1 hypersensitivity regions in CS15 craniofacial tissue but not with the TFAP2A ChIP-seq peaks in HeLa S3.

### Integration of nsCL/P GWAS data

Positional data of the hc-peaks were integrated with results from our recent nsCL/P GWAS meta-analysis^9^. This imputed dataset contains nsCL/P association data for ~ 8.01 million variants (meta_all_ as described in Ludwig et al.^9^). Briefly, this study included individuals of European (Bonn GWAS^17^: 399 cases and 1318 controls; Baltimore study^15^: 666 European case-parent trios) and of Asian ancestry (Baltimore study^15^: 795 Asian case-parent trios). The association P-values of the GWAS meta-analysis were not corrected for multiple testing. Only SNPs with an info score > 0.8 in the GWAS meta-analysis were retrieved, and pairs of SNPs in high linkage disequilibrium were identified using LD link (v.5; all populations). This selection of SNPs was intersected with topological associated domains in embryonal stem cells, as identified by Dixon et al.^35^, and information on the 45 nsCL/P risk loci as described in Welzenbach et al.^21^.

## Supplementary Information

Supplementary Information.

## Data Availability

The ChIP-seq datasets generated in the present study are available at the Gene Expression Omnibus (GEO) repository (accession numbers GSE169341 and GSE169342). The original GWAS datasets on which the GWAS meta-analysis of Ludwig et al.^9^ is based are available at Zenodo (https://doi.org/10.5281/zenodo.3724148; Bonn GWAS; Mangold et al.^17^) and dbGap (dbGaP: phs000094; Baltimore study; Beaty et al.^16^). References and online availability of datasets and tools employed in the project workflow are provided in Supplementary Table S15.
